# Global research trends in the tumor microenvironment of hepatocellular carcinoma: insights based on bibliometric analysis

**DOI:** 10.3389/fimmu.2024.1474869

**Published:** 2024-10-01

**Authors:** Hongmin Han, Ziyin Zhao, Mingyang He, Ge Guan, Junning Cao, Tianxiang Li, Bing Han, Bin Zhang

**Affiliations:** ^1^ Organ Transplantation Center, the Affiliated Hospital of Qingdao University, Qingdao, China; ^2^ Department of Hepatobiliary and Pancreatic Surgery, the Affiliated Hospital of Qingdao University, Qingdao, China

**Keywords:** hepatocellular carcinoma, tumor microenvironment, bibliometric, hotspots, trends

## Abstract

**Objective:**

This study aimed to use visual mapping and bibliometric analysis to summarize valuable information on the tumor microenvironment (TME)-related research on hepatocellular carcinoma (HCC) in the past 20 years and to identify the research hotspots and trends in this field.

**Methods:**

We screened all of the relevant literature on the TME of HCC in the Web of Science database from 2003 to 2023 and analysed the research hotspots and trends in this field via VOSviewer and CiteSpace.

**Results:**

A total of 2,157 English studies were collected. According to the prediction, the number of papers that were published in the past three years will be approximately 1,394, accounting for 64.63%. China published the most papers (n=1,525) and had the highest total number of citations (n=32,253). *Frontiers In Immunology* published the most articles on the TME of HCC (n=75), whereas, *Hepatology* was the journal with the highest total number of citations (n=4,104) and average number of citations (n=91). The four clusters containing keywords such as “cancer-associated fibroblasts”, “hepatic stellate cells”, “immune cells”, “immunotherapy”, “combination therapy”, “landscape”, “immune infiltration”, and “heterogeneity” are currently hot research topics in this field. The keywords “cell death”, “ferroptosis”, “biomarkers”, and “prognostic features” have emerged relatively recently, and these research directions are becoming increasingly popular.

**Conclusions:**

We identified four key areas of focus in the study of the TME in HCC: the main components and roles in the TME, immunotherapy, combination therapy, and the microenvironmental landscape. Moreover, the result of our study indicate that effect of ferroptosis on the TME in HCC may become a future research trend.

## 1 Introduction

Hepatocellular carcinoma (HCC) accounts for approximately 75-85% of primary liver cancers. Its incidence rate and mortality rate are rapidly increasing worldwide, with East Asia and Africa having the highest rates (1, 2). Although progress has been made in routine monitoring for the early detection of HCC, most patients are diagnosed in the late stage with limited treatment options and poor prognosis (3).

In recent years, the implementation scope of surgical and local regional treatment has been continuously expanding; however, approximately 50-60% of HCC patients will eventually receive systemic treatment, and unresectable HCC is more often treated with drugs targeting the tumor microenvironment (TME) (4, 5). With the progress of research, immune checkpoint inhibitors (ICIs) have been applied to the treatment of advanced HCC patients; however, the response rate is still low, and more than half of patients cannot benefit from ICIs (6), which may be related to the heterogeneity and complexity of the TME in HCC. The TME is defined as the complex and rich multicellular environment of tumor development and typically includes immune cells, stromal cells, extracellular matrix (ECM), other secretory molecules and lymphatic vascular networks (7). The dynamic interactions between tumor cells and the main components of the TME are crucial for generating heterogeneity in tumor cells, thus enhancing clonal evolution, promoting tumor cell growth and enhancing drug resistance in tumor cells (8, 9). Therefore, it is necessary to clearly define the TME of HCC, in order to identify new core treatment strategies, overcome drug resistance and improve patient prognosis.

Bibliometric analysis can qualitatively and quantitatively analyse publications in databases, and presents valuable information from all relevant literature in the form of visual graphs and tables, in order to reveal the development process, research hotspots and future trends of a certain field. With the application of ICIs and other drugs in the systematic treatment of advanced HCC, scholars from various countries have gradually provided more attention to the TME, and the number of papers related to TME in HCC has been steadily increasing each year. However, there has been no bibliometric analysis of the TME in HCC, and there is a lack of summary of research hotspots and prediction of development trends in this field. In this research, we offer insights into the TME literature related to HCC from the past two decades, drawing upon bibliometric analysis, and explore current research hotspots and emerging topics.

## 2 Methods

### 2.1 Data collection

**Figure 1:**
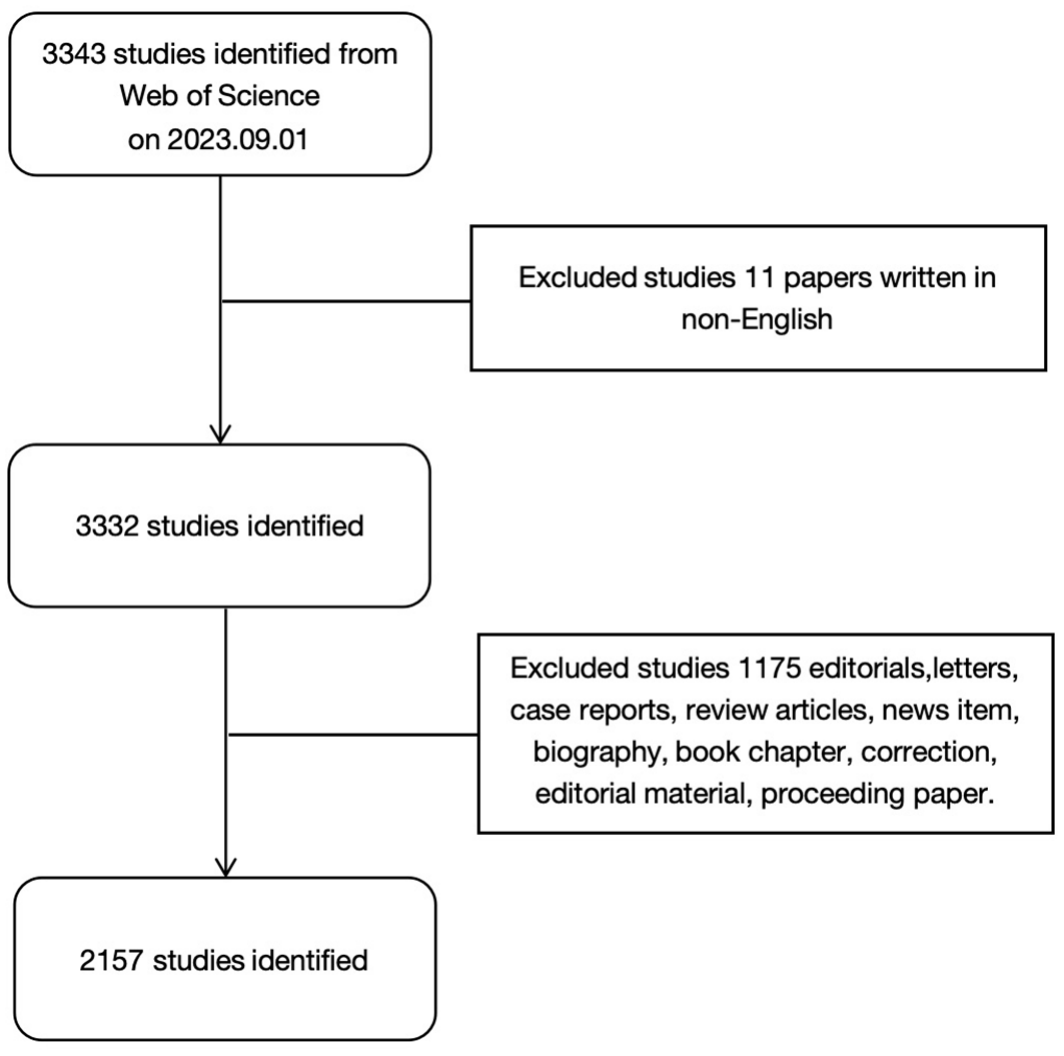
Flowchart of the search strategy.

We used the Web of Science database to search for relevant literature. To avoid bias caused by database updates, all of the literature and related data were downloaded manually on September 1, 2023. The search conditions were as follows. The publication date ranged from September 1, 2003 to September 1, 2023. We limited the search type to Article and the language to English. TS=(“liver neoplasm *” OR “hepatic cancer” OR “liver cancer” OR “hepatic cancer *” OR “hepatic cancer *” OR “hepatic cancer *” OR “hepatic cell cancer *”) AND TS=(“Tumor Microenvironment *” OR “Cancer Microenvironment *” OR “Cellular Microenvironments” OR “Cell Microenvironment *”). The retrieval process is shown in **Figure 1**.

### 2.2 Analysis tools and methods

VOSviewer and CiteSpace are classic bibliometric analysis software. Unlike other visual mapping programs, VOSviewer pays special attention to the graphical representation of bibliometric maps, and is suitable for displaying large bibliometric maps in an easy to interpret manner (10). However, the purpose of Citespace is to assist clinical researchers in generating domain-specific datasets, aiming to facilitate the analysis of emerging trends in selected fields (11). They can support various types of bibliometric research, including co-citation analysis, co-occurrence analysis, and burst analysis. In this study, we first completed the co-authorship analysis and co-occurrence analysis by using VOSviewer1.6.18, and established a visual map. In addition, we also completed a descriptive analysis of publication year, countries, institutions, journals, authors, and references. Finally, we used CiteSpace 6.2.R6 to perform the timezone map analysis and burst analysis on keywords.

## 3 Results

### 3.1 Annual publications

**Figure 2:**
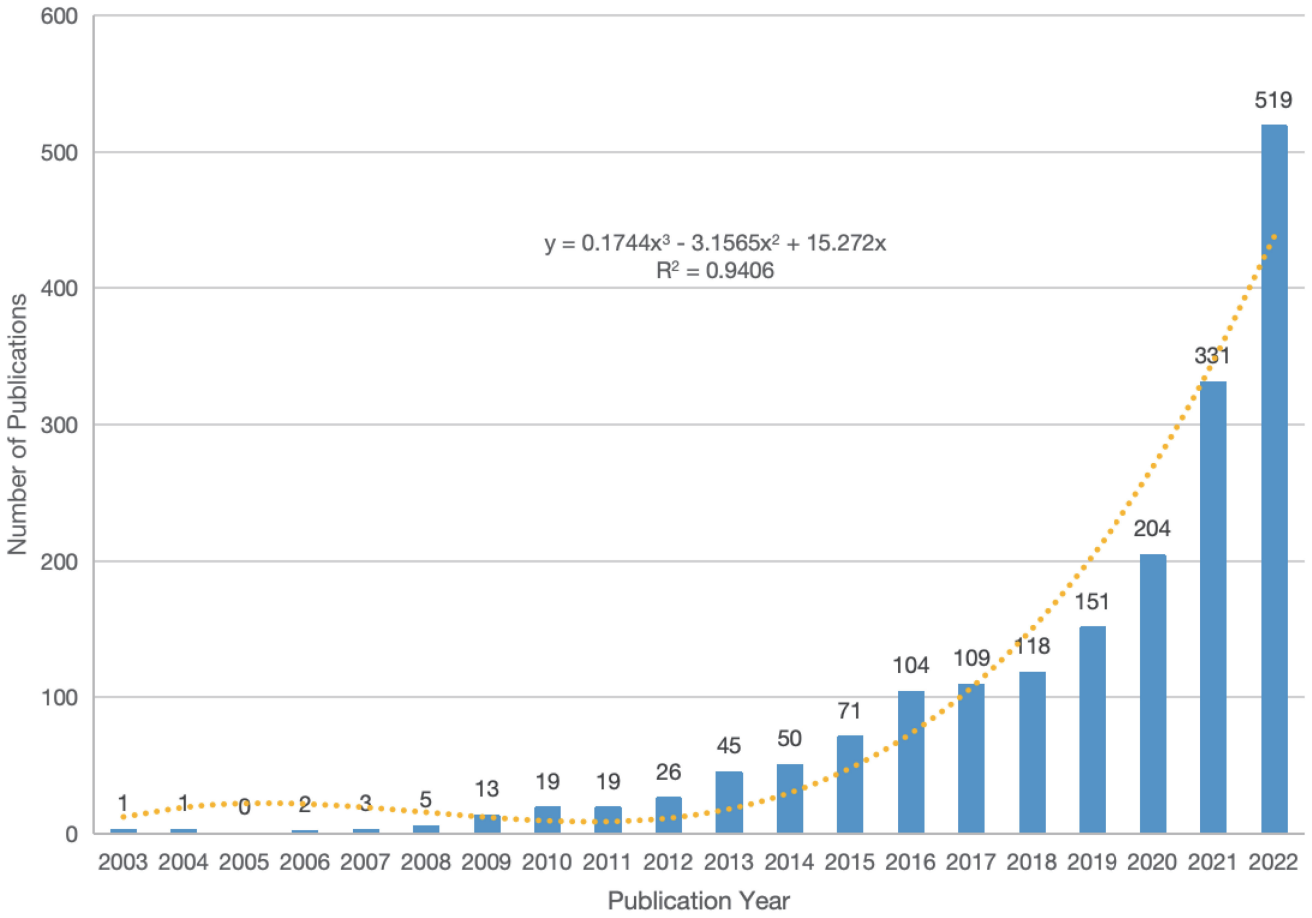
Annual number and growth trends of publications from 2003 to 2022.

We retrieved a total of 2,157 articles related to the TME of HCC and included them in the final study analysis. The trend of publications from 2003 to 2022 is shown in **Figure 2**, thus demonstrating an overall upwards trend. Via linear regression analysis, we predict that the total number of publications in 2023 will reach approximately 544. According to this prediction, the total number of publications from 2021 to 2023 will be approximately 1,394, which accounts for 64.63%. This indicates that research related to the TME of HCC has received increasing amounts of attention.

### 3.2 Analysis of journals

Between 2003 and 2023, a total of 580 academic journals with published articles related to the TME of HCC were identified. We list the top 10 journals with the most published articles and related impact indicators in **Table 1**. The most publications originated from *Frontiers in Immunology* (n=75). *Hepatology* is the journal with the highest total number of citations (n=4,104), and its impact factor (IF=13.5) is also the highest, thus indicating that the quality of the articles in this journal is relatively high and that it is authoritative in the research field. The journals in the *Frontiers* series had the highest average publication year, thus indicating that this series of journals has shown great interest in the field and that the number of related articles has shown rapid growth in recent years.

**Table 1:** Top 10 journals in terms of publication volume.

Rank	Journal	Counts	Citations	Avg. pub. year	IF
1	Frontiers In Immunology	75	729	2021.71	7.3
2	Frontiers In Oncology	67	538	2021.67	4.7
3	Hepatology	45	4104	2018.47	13.5
4	Cancer Letters	42	1811	2019.00	9.7
5	Frontiers In Genetics	42	166	2021.79	3.7
6	Journal For Immunotherapy Of Cancer	38	1005	2021.24	10.9
7	Oncotarget	38	1324	2016.16	5.1
8	Aging-US	34	320	2020.89	5.2
9	International Journal Of Molecular Sciences	31	281	2021.23	5.6
10	Scientific Reports	30	898	2018.63	4.6

### 3.3 Analysis of countries

Fifty-four countries throughout the world have participated in research related to the TME of HCC, and we list the top 10 countries with the greatest number of publications in **Table 2**. China is the most productive country (n=1,525, 70.7%). Moreover, China is also the country with the highest total number of citations (n=32,253, 61.7%). The number of publications and the total number of citations from China both accounted for more than 60%, which indicated that Chinese scholars have made significant contributions to research in this field.

**Table 2:** Top 10 countries in terms of the number of publications.

Rank	Country	Counts	Citations	Avg. citations
1	CHINA	1525	32253	21
2	USA	182	8206	45
3	JAPAN	76	2434	32
4	SOUTH KOREA	73	1635	22
5	ITALY	45	1203	27
6	GERMANY	38	839	22
7	SINGAPORE	25	950	38
8	INDIA	21	536	26
9	FRANCE	18	907	50
10	EGYPT	15	277	19

**Figure 3:**
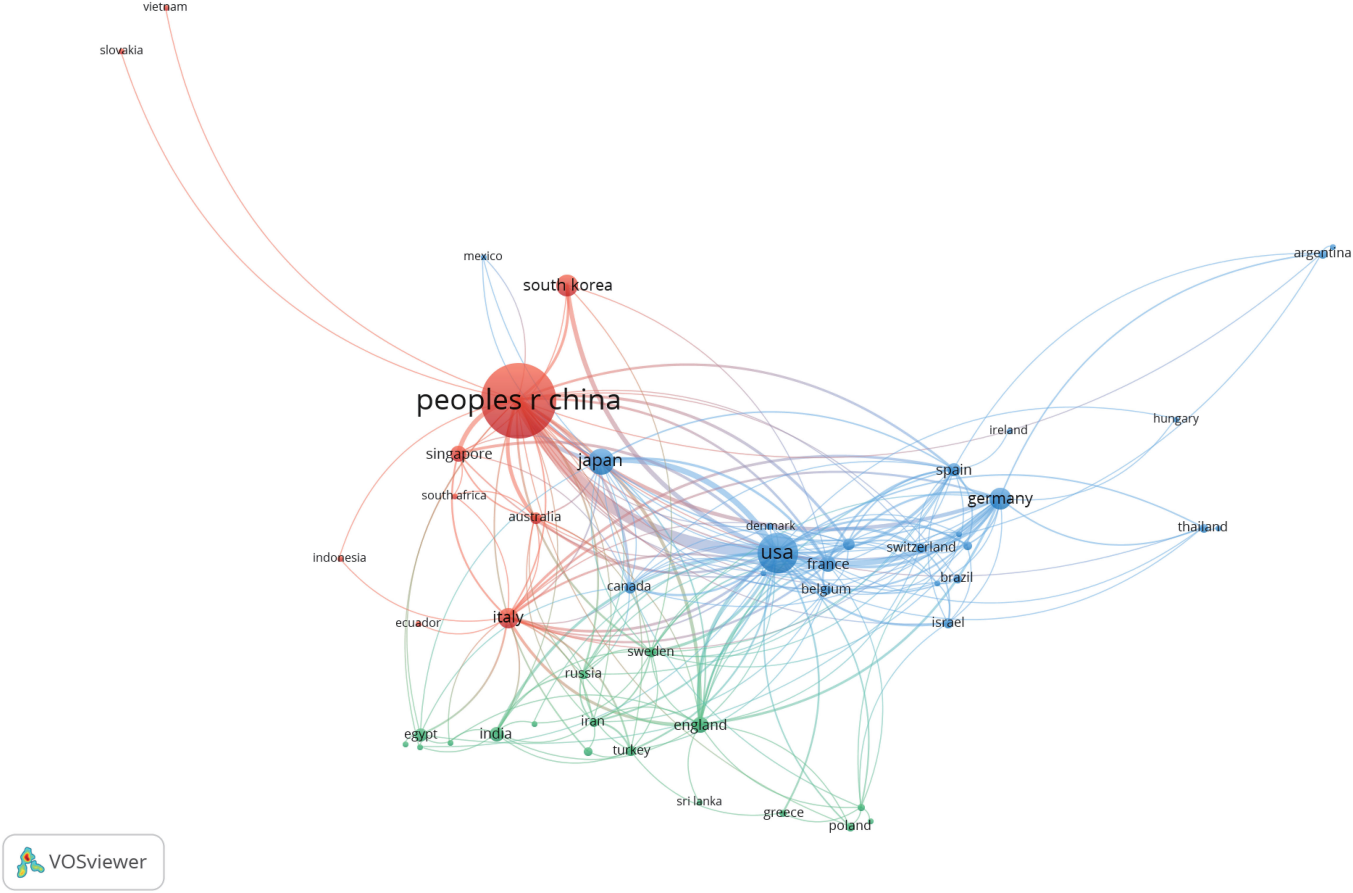
Cooperation between countries based on VOSviewer.

In addition, we conducted a co-authorship analysis of 54 countries and regions by using VOSviewer. As shown in **Figure 3**, both China and the United States cooperate with 34 countries and regions, followed by Italy with 23. China and the United States have a significant influence on international cooperation.

### 3.4 Analysis of institutions

According to our data analysis of the collected literature, a total of 2,081 institutions participated in the publication of papers related to the TME of HCC, and the top 10 institutions in terms of publication volume are shown in **Table 3**. These 10 institutions originated from China, with Fudan University having the highest number of publications (n=135). Fudan University also has the highest total number of citations (n=4,541), thus demonstrating its authority and outstanding contribution in this research field. In addition, Zhengzhou University has the highest average publication year and has good research potential in this field.

**Table 3:** Top 10 institutions with the greatest number of publications.

Rank	Institution	Counts	Citations	Avg. pub. year
1	Fudan University	135	4541	2019.82
2	Sun Yat-sen University	117	4210	2019.83
3	Zhejiang University	82	1864	2020.34
4	Shanghai Jiao Tong University	75	1591	2020.20
5	Huazhong University of Science and Technology	73	1664	2020.01
6	Chinese Academy of Sciences	61	2149	2019.66
7	Southern Medical University	58	1305	2020.97
8	Xi’an Jiao Tong University	47	1016	2019.72
9	Zhengzhou University	46	600	2021.07
10	Sichuan University	45	613	2020.44

**Figure 4:**
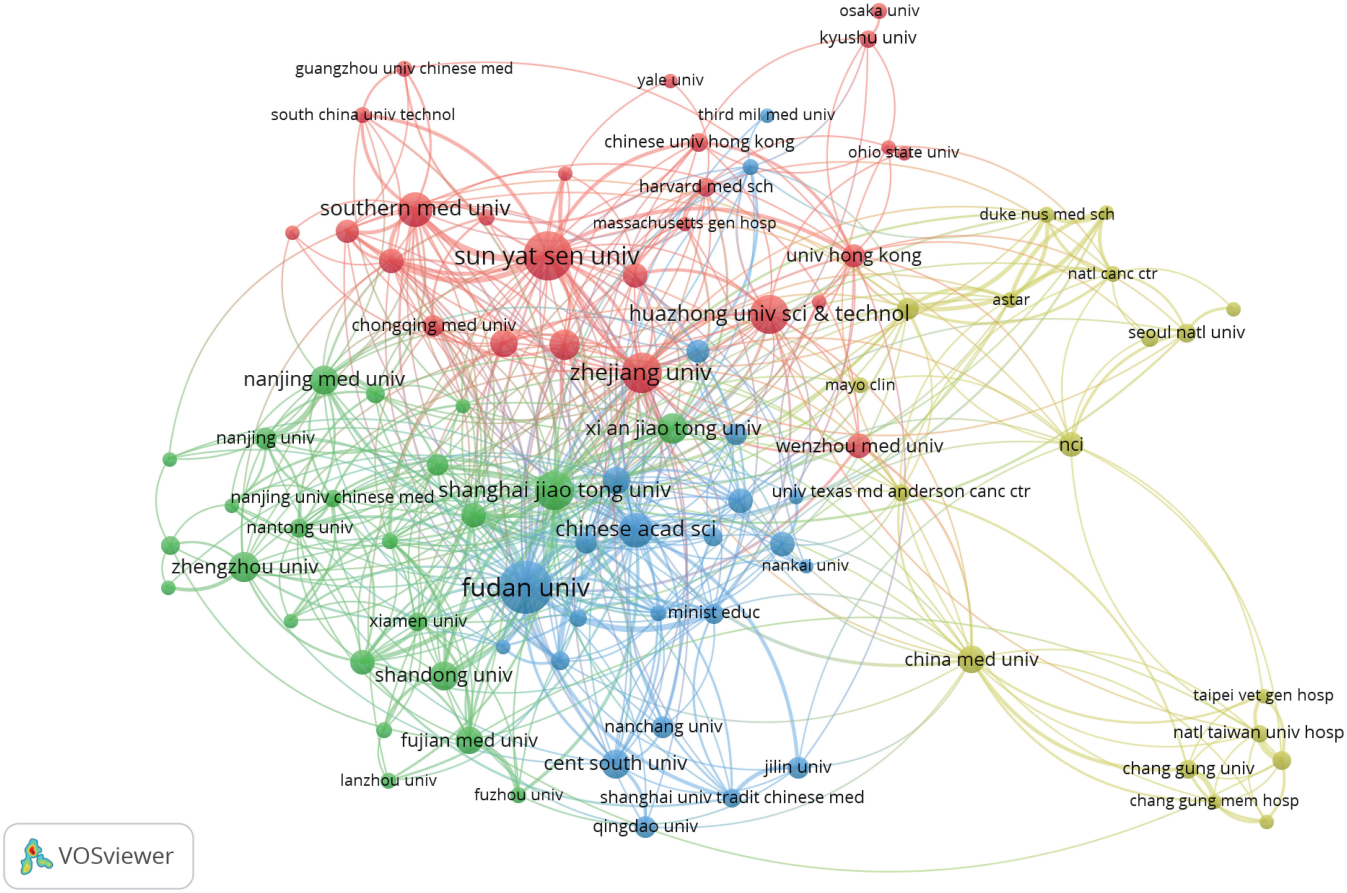
Collaboration between institutions based on VOSviewer.

We set the minimum number of publications to 10, identified 92 high-yield institutions from 2,081 institutions, and conducted a co-authorship analysis on these 92 high-yield institutions by using VOSviewer. As shown in **Figure 4**, Shanghai Jiao Tong University may be the most influential institution in terms of collaboration, wherein it collaborated with 40 high-yield institutions.

### 3.5 Analysis of authors

**Table 4** shows the top 10 authors in terms of publication volume in research related to the TME of HCC. Fan Jia and Zhou Jian are the authors with the greatest number of publications, each with 36 articles. These two scholars also have the most total citations (Fan Jia [n=2,127] and Zhou Jian [n=1,886]), thus indicating that their research results are widely recognized and may be authoritative representatives in the field.

**Table 4:** Top 10 authors with the greatest number of publications.

Rank	Author	Counts	Citations	Avg. citations
1	Fan, Jia	36	2127	59
2	Zhou, Jian	36	1886	52
3	Zheng, Li-min	19	1425	75
4	Li, Jing	16	319	20
5	Zhang, Jian	16	559	35
6	Gao, Qiang	15	759	51
7	Yang, Yang	15	1093	73
8	Li, Yan	14	488	35
9	Wang, Wei	13	126	10
10	Wu, Yan	13	1080	83

**Figure 5:**
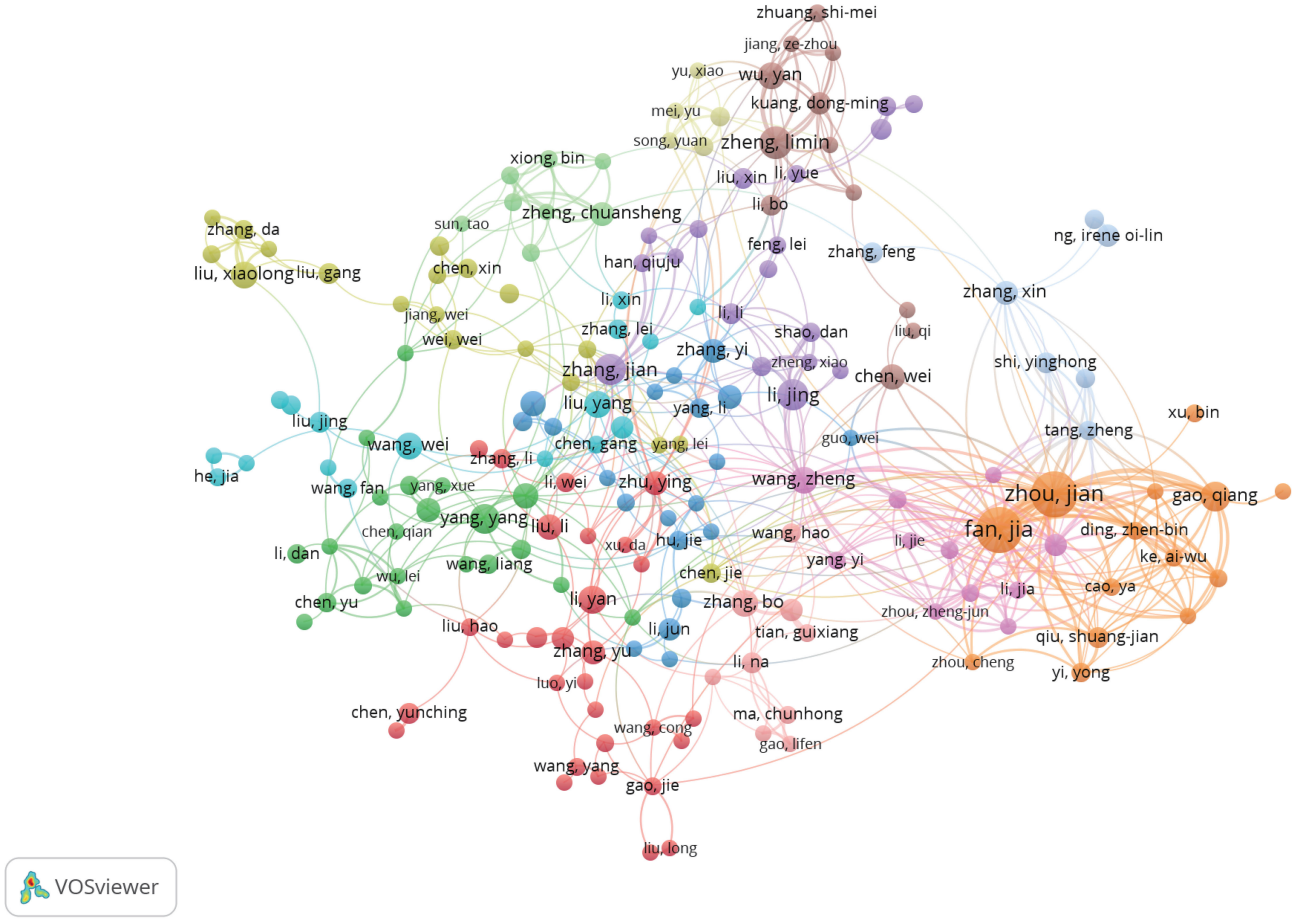
Collaboration between authors based on VOSviewer.

We set the minimum number of publications to 5, identified 219 high-yield authors from 10,641 authors, and conducted a co-authorship analysis on these 219 high-yield authors by using VOSviewer. Fan Jia and Zhou Jian collaborated with 35 high-yield authors, wherein they ranked first (**Figure 5**).

### 3.6 Highly cited literature

We list the top 10 articles with the greatest number of citations in **Table 5**, of which 5 are from China, 3 are from the United States, and the remaining 2 are from Japan and Italy. Chinese scholars have made outstanding contributions to and influence on the field of the TME in HCC.

**Table 5:** The top 10 most cited references in the field of the TME of HCC.

Rank	Title	Pub. year	Citations	Country
1	Phase Ib Study of Lenvatinib Plus Pembrolizumab In Patients With Unresectable Hepatocellular Carcinoma	2020	552	USA
2	Tumor-Associated Neutrophils Recruit Macrophages and T-regulatory Cells to Promote Progression of Hepatocellular Carcinoma and Resistance to Sorafenib	2016	475	CHINA
3	Carcinoma-associated fibroblasts promote the stemness and chemoresistance of colorectal cancer by transferring exosomal lncrna H19	2018	408	CHINA
4	Tumor-Specific T Cell Dysfunction Is a Dynamic Antigen-Driven Differentiation Program Initiated Early During Tumorigenesis	2016	391	USA
5	FAP Promotes Immunosuppression by Cancer-Associated Fibroblasts in the Tumor Microenvironment via STAT3-CCL2 Signaling	2016	383	CHINA
6	Tim-3/galectin-9 signaling pathway mediates T-cell dysfunction and predicts poor prognosis in patients with hepatitis B virus-associated hepatocellular carcinoma	2012	360	CHINA
7	Probiotics modulated gut microbiota suppresses hepatocellular carcinoma growth in mice	2016	355	CHINA
8	Neutrophil-lymphocyte ratio reflects hepatocellular carcinoma recurrence after liver transplantation via inflammatory microenvironment	2013	352	JAPAN
9	CD90+liver cancer cells modulate endothelial cell phenotype through the release of exosomes containing H19 lncrna	2015	341	ITALY
10	DNA damage-mediated induction of a chemoresistant niche	2010	332	USA

It is worth mentioning that the article “Phase Ib Study of Lenvatinib Plus Pembrolizumab In Patients With Unresectable Hepatocellular Carcinoma”, which ranked first in citation count, was published in July 2020 and increased to the top spot in just three years. These results are highly significant, and the attention and expectations of global researchers are gradually shifting towards combination therapy.

### 3.7 Keyword analysis

**Figure 6:**
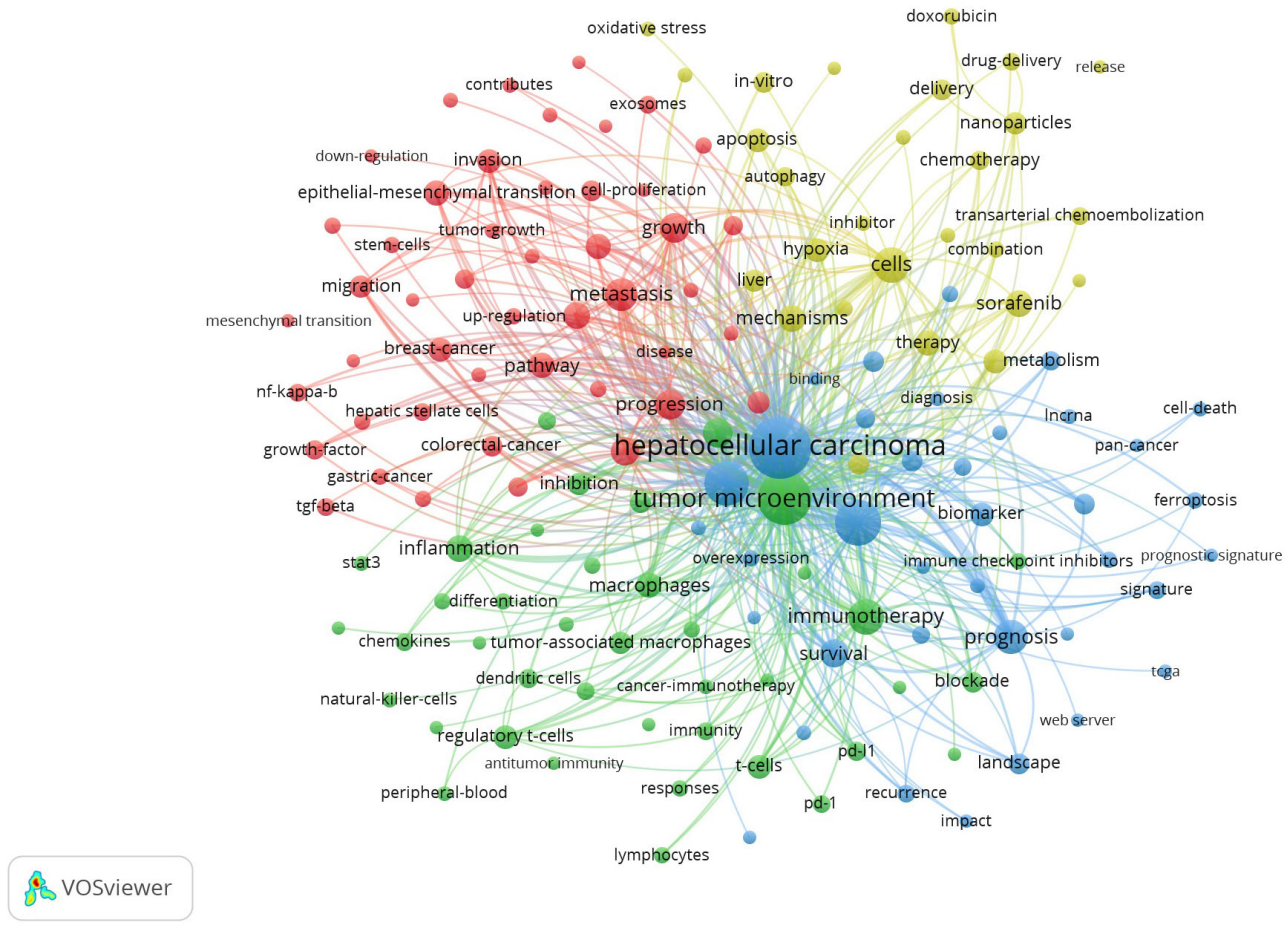
Keyword cluster analysis and co-occurrence analysis based on VOSviewer.

We extracted a total of 6,846 keywords from these 2,157 articles. To more intuitively and quickly obtain the frontiers and hotspots of research related to the TME of HCC, We set the minimum number of keyword occurrences to 20, conducted co-occurrence analysis on 151 high-frequency keywords that met the conditions, and constructed a co-occurrence network diagram of high-frequency keywords. As shown in **Figure 6**, these high-frequency keywords are mainly divided into four clusters, as represented by different colours. By integrating the keyword frequency within each cluster and the average co-occurrence year, we determined the research focus for each cluster. The red clusters represent the main components and roles in the TME of HCC, such as “extracellular matrix”, “cancer-associated fibroblasts” and “hepatic stellate cells”; the green clusters represent immunotherapy for HCC, such as “immunotherapy”, “PD-1” and “immune cells”; the yellow clusters represent combination therapy for HCC, such as “combination therapy”, “targeted therapy” and “resistance”; and the blue clusters represent the microenvironmental landscape of HCC, such as “landscape”, “immune infiltration” and “heterogeneity”.

**Figure 7:**
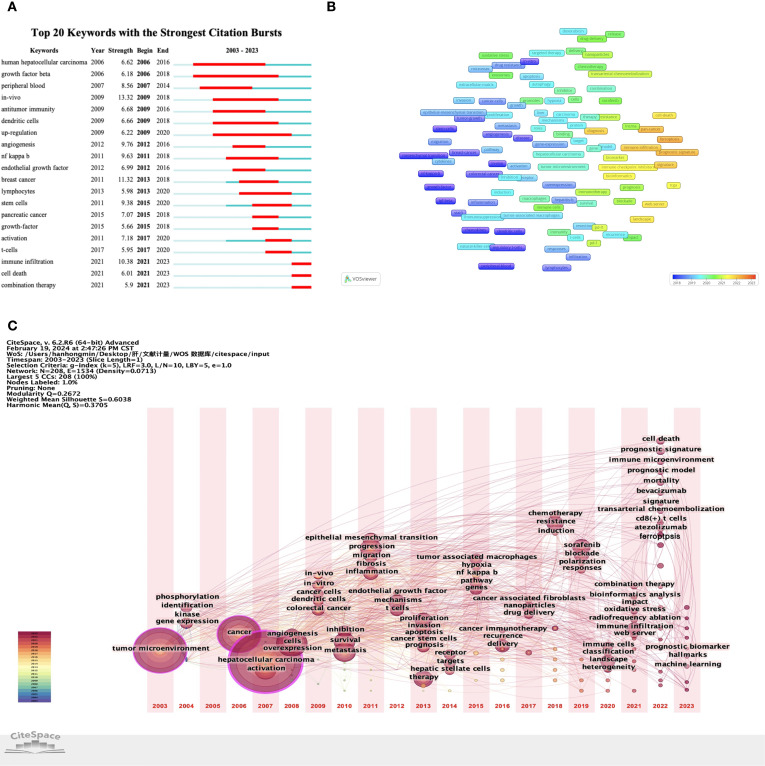
Keyword analysis based on VOSviewer and CiteSpace. **(A)** Analysis of keyword bursts. **(B)** The co-occurrence overlay of keywords. **(C)** Timezone map of keywords.

In addition, we also found that the keyword “cell death” has seen a spike in citations over the last three years (**Figure 7A**). Related keywords such as “ferroptosis”, “biomarker” and “diagnostic signature” appeared around 2022 (**Figures 7B, C**). It shows that these research directions have received increasing attention in recent years and may become the research hotspots in the future.

## 4 Discussion

### 4.1 General information

In this study, we identified literature studies on the TME of HCC over the past 20 years in the Web of Science database (2003-2023). After excluding studies that did not meet the screening criteria, a total of 2,157 English studies published in 580 academic journals and from 2,081 institutions in 54 countries were included.

The number of publications related to the TME of HCC has been increasing annually, especially in the past three years, when the number of publications exceeded 300. According to linear regression analysis, the total number of publications from 2021 to 2023 will reach approximately 1,394, accounting for 64.63%. We believe that the main reason for this explosive growth involves the breakthrough of immunotherapy in HCC. In addition, it is worth noting that among the top ten cited articles, “Phase Ib Study of Lenvatinib Plus Pembrolizumab In Patients With Unresectable Hepatocellular Carcinoma” is the only clinical trial study, and this article risen to the top of the list in just three years since its publication. The focus and expectations of global researchers are gradually shifting towards combination therapy, especially the combination of ICIs and other drugs.

Based on the general information distribution of the collected literature, China ranks first in the world, whereas the United States and Japan rank second and third respectively. The point worth emphasizing is that the number of publications and the total number of citations from China exceed those from other countries combined (**Table 2**), and the top 10 research institutions and scholars in terms of publications are all from China (**Tables 3** and **4**). China has invested a lot in research in this field. We believe that the potential reason is that the five-year survival rate of Chinese patients with liver cancer is low, less than 15% (12), and new cases account for approximately half of the global total (13). In an effort to enhance survival rates and improve prognoses, Chinese institutions and scholars are dedicated to research in this field. Among them, Fudan University’s contribution is particularly outstanding, and the two scholars with the highest publication volume (Fan Jia and Zhou Jian) also originate from this institution. Fudan University can be considered representative of the TME in HCC.

### 4.2 Hotspot directions

Through the analysis of high-frequency keywords, we have summarized four hotspot directions in the field of the TME in HCC: (1) the main components and roles in the TME; (2) immunotherapy; (3) combination therapy; and (4) the microenvironmental landscape.

#### 4.2.1 The main components and roles in the TME of HCC

##### 4.2.1.1 Immune cells

Cytotoxic T (CD8+) cells are key effector cells in anti-tumor immunity, and their dysfunction is the main factor in HCC immune escape (14). Previous studies have shown that the density of CD8+ T cells infiltrating tumors is associated with longer survival in HCC patients (15), and the reduction in the depletion and dysfunction of CD8+ T cells is one of the current strategies for the treatment of tumors. Regulatory T (T_reg_) cells are key cells mediating immune suppression associated with HCC, and this immune suppression function may be mediated by T_reg_ cells secreting CD10, transforming growth factor-β (TGF-β), IL-10 and IL-35 (16, 17). Analysis of human HCC samples demonstrated that the accumulation of T_reg_ cells can reduce the infiltration of CD8+ T cells in HCC tumor areas and is associated with high mortality in patients (18).

Tumor-associated macrophages (TAMs) are key players in tumor-associated inflammation and play an important role in the microenvironment by inducing M1 and M2 polarization (19). In HCC, M1 can inhibit tumor progression through various mechanisms, whereas M2 can promote the progression of HCC by secreting a variety of cytokines and exosomes, and M2 has been found to be closely related to tumor stem cells in HCC (20).

Tumor-associated neutrophils (TANs) are the main component of the TME, and inappropriate activation may cause inflammatory disorders in the TME, thus leading to the occurrence and development of HCC (21). TANs also exhibit two different anti-tumor (N1) and pro-tumor (N2) phenotypes in HCC (22). TGF-β plays a major role in the plasticity of TANs, thus driving the acquisition of N2 TANs. However, blockage of TGF-β leads to the recruitment and activation of N1 TANs, which have anti-tumor effects (23). Therefore, the functional regulation and phenotypic transformation of TANs may be potential therapeutic strategies for treating HCC.

Natural killer cell (NK cell) play an important role in the immune response against HCC, and their cytotoxic effects on tumor cells have also been confirmed (24). Decreased number of NK cell infiltration, decreased cell activity, or the absence of NK cell characteristics in HCC are all associated with poor patient prognosis (25). In addition, molecular targeted drugs (such as sorafenib) can trigger an anti-tumor response in liver NK cells, thus resulting in increased cytotoxicity (26). A greater number of enriched NK cells in HCC corresponds to a greater therapeutic effect of sorafenib (27).

##### 4.2.1.2 Nonimmune components

In HCC, cancer-associated fibroblasts (CAFs) originate from activated hepatic stellate cells, are closely related to the TME, and affect the progression of HCC (28). CAFs have a regulatory effect on the immune cell function in the TME and can induce other immune cells to acquire special phenotypes, thereby promoting the malignant behaviour of HCC and tumor immunosuppression (29, 30). In addition, CAFs can also induce resistance to tyrosine kinase inhibitors (TKIs) through paracrine pathways, thus inhibiting the sensitivity of HCC to sorafenib and lenvatinib (31). Scholars have summarized the potential therapeutic strategies of CAFs in HCC: 1) specific eradication of CAFs, 2) prevention of precursor cell from conversion to CAFs and inhibition of the paracrine function of CAFs, and 3) targeting of CAFs-mediated crosstalk (32).

The disordered arrangement of the ECM in the TME of HCC contributes to the immune escape of tumor cells; moreover, it promotes rapid proliferation, angiogenesis, and metastasis, as well as reduces the apoptosis of tumor cells. This “disorganization” is reflected in the degradation and remodelling of the ECM (33). Fan et al. (32) suggested that the secretion of CAFs can regulate ECM remodelling, while ECM degradation and collagen fibre arrangement help promote CAF activation, they form a feedback loop in the TME to jointly promote the development of HCC. In addition, the increased matrix stiffness of the ECM can promote the proliferation, epithelial-mesenchymal transition (EMT) phenotype, and stemness characteristics of HCC cells (34).

As a component of the TME, mesenchymal stem cells (MSCs) have obvious tropism for tumor sites (35). Due to this tropism, MSCs (or their driven extracellular vesicles) can be used as drug delivery vehicles to target anti-HCC treatment, thus improving therapeutic efficacy and reducing side effects (36–38). Therefore, MSCs are expected to become a promising systemic delivery tool for the treatment of HCC. However, the role of MSCs in the development of HCC remains controversial, potentially exerting both pro-tumor and anti-tumor effects. The mechanism of MSCs in the TME still requires further research.

#### 4.2.2 Immunotherapy

For the systematic treatment of HCC, TKIs such as sorafenib, lenvatinib, regorafenib, and cabozantinib have been approved for first-line and second-line treatment of advanced HCC and can improve patient survival (39, 40). However, the overall survival (OS) of patients with advanced HCC is still very short. With the increased in-depth research in recent years, immunotherapy that can target TME has provided new directions for the treatment of HCC and has become a component of systemic therapy for HCC. Immunotherapy strategies include ICIs, adoptive cell therapies (ACTs), vaccines and oncolytic viruses. Currently, the main application category is ICIs, especially ICIs that block PD-1 or PD-L1.

##### 4.2.2.1 Immune checkpoint inhibitors

ICIs prevent T-cell inactivation by blocking the interaction between checkpoint proteins and their ligands, thereby exerting anti-tumor effects (41). Therefore, ICIs reshape cancer treatments, including those for HCC, by exhibiting anti-tumor activity and prolonging patient survival (42).

The application of ICIs in advanced HCC treatment is tortuous. Nivolumab and pembrolizumab, which are two PD-1 inhibitors, were once accelerated and approved by the US Food and Drug Administration (FDA) for the treatment of HCC patients with sorafenib progression or intolerance. Although the objective response rate (ORR) and safety of these two ICIs after use have been confirmed, they failed to prove their superiority in terms of OS and other aspects, and the prespecified endpoints were not reached (5, 42). Therefore, indications for nivolumab monotherapy for HCC patients were withdrawn from the US market after consultation with the FDA. Pembrolizumab significantly prolongs OS and progression-free survival (PFS) in the Asian subgroup and is recommended for second-line treatment of advanced HCC in Asian countries such as China (43, 44).

At present, there are few clinical studies of ICI monotherapy for the treatment of advanced HCC. We expect that more new ICIs can be applied in clinical practice to benefit patients.

##### 4.2.2.2 Adoptive cell therapy

ACT targets malignant tumors by infusing activated and expanded autologous or allogeneic immune effector cells into the body, among which chimeric antigen receptor T-Cell immunotherapy (CAR-T) is the most common and important (45). Clinical trials using glypican-3 (GPC3) as a target are the most common CAT-T treatment for HCC, and its anti-tumor activity and safety have been preliminarily determined (46, 47); however, most of these trials are phase I trials that require further research and confirmation in larger-scale trials.

##### 4.2.2.3 Oncolytic viruses

Oncolytic virus (OV) is a type of natural or recombinant virus that selectively infects cancer cells and replicates within them, thus causing cell lysis without affecting normal tissue (48). In HCC, OVs can not only kill tumor cells through their own action but also induce immune responses to mediate anti-tumor therapy (49, 50). In addition, the targeting of OV to HCC through the use of MSCs as carriers can improve safety and anti-tumor effects (36, 38).

#### 4.2.3 Combination therapy

In the systematic treatment of HCC, to enhance the responsiveness of tumor to immune checkpoint suppression, new combination therapy strategies such as ICIs combined with antiangiogenic drugs, other molecular targeted therapies and complementary ICIs, are being developed and have shown good activity (17).

Currently, the representative combination of ICIs and antiangiogenic drugs is atezolizumab plus bevacizumab, which has been applied as a first-line treatment for advanced HCC in clinical practice. In the IMbrave150 trial, the median OS of the atezolizumab plus bevacizumab group was 5.8 months longer than that of the sorafenib group, with an ORR of 30.0% (51). Due to its significant clinical benefits, this strategy is considered to be a new milestone.

In addition, due to the fact that lenvatinib can improve the clinical benefits of PD-1 antibodies by enhancing the anti-tumor immune response, Finn and Llovet et al. conducted a study on the combination of lenvatinib plus pembrolizumab as a first-line drug for the treatment of advanced HCC (52). In a single-arm phase Ib study (KEYNOTE 524), the ORR of RECIST v1.1 was 36.0%, with a median PFS and OS of 8.6 and 22.0 months, respectively, which were encouraging results. Subsequently, in a phase III study (LEAP-002), lenvatinib plus pembrolizumab was compared with lenvatinib plus placebo; the ORR of RECIST v1.1 was 26.1% vs. 17.5%, and the median PFS and OS were 8.2 vs. 8.0 months and 21.2 vs. 19.0 months, respectively (52, 53). Although the survival time was longer than that of the control group, the research results do not support changes in clinical practice because the findings did not reach prespecified significance.

The combination of nivolumab and ipilimumab was the first approved dual immunotherapy regimen for the treatment of advanced HCC (CheckMate 040). Due to its controllable safety and high ORR (RECIST v1.1: 31-32%), the FDA has accelerated the approval of its use as a second-line treatment in patients who have previously received sorafenib treatment (54, 55). The subsequent phase III trial will compare nivolumab plus ipizumab as a first-line treatment with lenvatinib or sorafenib (CheckMate 9DW), and we anticipate more surprising results. In addition, the HIMALAYA trial is considered to be a milestone in dual-immune combination strategies. Compared with sorafenib, durvalumab plus tremelimumab showed significant clinical benefits as a first-line treatment, with a median OS of 16.4 months vs. 13.8 months and an ORR of 20.1% vs. 5.1%, respectively (56).

As researchers focus on combination therapy strategies, an increasing number of new strategies are being developed. In addition to the combination of the abovementioned drugs, clinical trials of drugs combined with transarterial chemoembolization (TACE) (57), radiation therapy (58) and other methods for the treatment of advanced HCC are also gradually increasing. We also look forward to the application of more new combination therapy strategies that can benefit patients in clinical practice.

#### 4.2.4 The microenvironmental landscape of HCC

HCC has a complex and highly heterogeneous ecosystem. The microenviromental landscape of HCC was visualized through single-cell RNA sequencing, spatial transcriptomics and bioinformatics to analyse the heterogeneity and other biological information of HCC.

Via landscape analysis of the TME in HCC, scholars have found that the microenvironment of the tumor boundary area is particularly special. The main features of this region include increased activation of hypoxia response pathways, angiogenesis, immune escape and EMT, a strong inflammatory response, severe hepatocyte damage and a local immunosuppressive microenvironment (59). There are also a large number of intermediate-state cells with molecular characteristics between those of tumor cells and normal cells in this region (60). Therefore, it is speculated that the tumor boundary area can determine the risk of tumor invasion and the prognosis of patients, and may become a targeted region for precise treatment strategies.

Due to the heterogeneity of HCC, Wang et al. classified HCC patients into three subtypes based on immune cell infiltration: S1 is characterized by a “hot tumor” with high infiltration of immune cells and the best prognosis; S2 is characterized by a “cold tumor” due to its low immune infiltration rate; and S3 is characterized by an “immunosuppressive tumor” with high expression of immunosuppressive genes (CTLA4 and TIGIT) and the worst prognosis (61). In addition, Xue et al. classified the TME of HCC patients into five subtypes based on the differential enrichment of cells in tumor tissue, including immune activation, myeloid or stromal cell-mediated immunosuppression, immune rejection, and the immune persistence phenotype (62). There are many studies of this type; however, there is no consensus on the results. The ability to accurately classify HCC based on the TME and select appropriate treatment strategies for different subtypes of HCC could improve the effectiveness of systematic treatment.

The microenvironmental landscape of HCC can also identify stimulating factors and related mechanisms at different stages of tumor occurrence, development, and metastasis, which is helpful for identifying effective immunotherapy targets and biomarkers (63–65). In summary, with the use of advanced scientific technology and tools, more microscopic and detailed biological information can be displayed, thus helping researchers to gain a deeper understanding of the TME and providing a foundation for subsequent research.

Collectively, the four current research hotspots encompass both clinical and basic research. Generally, clinical research focuses on ICIs, whether as monotherapy or in combination therapy; the basic research is the landscape display based on the main components of TME. Consequently, researchers are afforded more options, based on the nature of their work and individual strengths.

### 4.3 Research trends

Through keyword analysis of the TME in HCC, we found that the keyword “cell death” has seen a spike in citations over the last three years, and related keywords such as “ferroptosis”, “biomarker” and “diagnostic signature” have also emerged relatively recently, thus indicating that these research directions are becoming increasingly popular. Combined with the current research hotspots, we speculate that the effect of ferroptosis on TME in HCC may become a future research trend.

Increasing evidence indicates that the activation of ferroptosis may effectively inhibit the growth of HCC cells (66). Several studies have constructed prognostic signature models for HCC based on ferroptosis-related long noncoding RNAs or genes, which can predict tumor heterogeneity, immunotherapy response, immune suppression status, and other characteristics (67–70). Furthermore, scholars have elucidated the relationship between immune cells and ferroptosis, and there are many underlying mechanisms. Changes in these mechanisms can significantly affect innate immunity and adaptive immunity (71, 72), which seems to indicate that ferroptosis is closely related to the TME.

In recent years, studies have shown that the inhibition of APOC1 can reverse the transformation of M2 TAMs to M1 TAMs through the ferroptosis pathway, thereby inhibiting the development of HCC (73). In addition, the inhibitor iFSP1 of ferroptosis suppressor protein 1 can not only induce ferroptosis in HCC but also increase the infiltration of immune cells such as M1 macrophages, dendritic cells, and CD8+ T cells in tumors (74). This means that ferroptosis can synergize with anti-tumor immune responses. However, ferroptosis seems to have an antagonistic effect on the anti-tumor immune response. The anti-tumor effects of immune cells are affected by ferroptosis, thus leading to a decrease in anti-tumor activity, and ferroptosis can also trigger the infiltration of immunosuppressive cells into tumors (75–77). Therefore, an exploration of the mechanism of ferroptosis in the TME and the identification of candidate genes and pathways that can induce ferroptosis in HCC while preserving anti-tumor immune cell function may be the focus of subsequent research.

In conclusion, ferroptosis is closely related to the TME and has synergistic and antagonistic effects on anti-tumor immune responses in HCC. These two effects seem contradictory, but they are very common and reasonable in the process of biological development. The reason for this effect may be that the mechanism of ferroptosis in the TME is not clear. Therefore, it is necessary to understand the mechanism of ferroptosis in the TME of HCC, and on this basis to explore new diagnostic biomarkers and effective therapeutic targets, which may be a potential therapeutic strategy.

## 5 Conclusions

In summary, this study used bibliometric analysis methods to summarize the current research hotspots and trends in the field of the TME in HCC. Research has shown that the TME is crucial for the diagnosis and treatment of HCC. At present, the research hotspots in the TME of HCC include four aspects: the main components and roles in the TME, immunotherapy, combination therapy and the microenvironmental landscape. By combining current research hotspots and emerging keywords, the effect of ferroptosis on the TME in HCC may become a future research trend.

## 6 Limitations

This study had some inevitable limitations. Firstly, we included only the core studies in the Web of Science database, and the language was limited to English. Secondly, we screened all of the literature on September 1, 2023, and the Web of Science database was still updated during the completion of this study. Therefore, some relevant articles may have been excluded. Thirdly, some subjective speculations cannot be completely avoided, which leads to the lack of convincing in some conclusions. Lastly, studies recently published in prestigious journals may be overlooked for their significance due to low citation rates. We believe that there is a need for a new strategy to continuously track research in this field and periodically summarize its updated data to narrow limitations and make research more persuasive and instructive.

## Data Availability

The original contributions presented in the study are included in the article/supplementary material. Further inquiries can be directed to the corresponding authors.
